# Measuring vincristine-induced peripheral neuropathy in children with cancer: validation of the Dutch pediatric–modified Total Neuropathy Score

**DOI:** 10.1007/s00520-019-05106-3

**Published:** 2019-11-16

**Authors:** S. M. Schouten, M. E. van de Velde, G. J. L. Kaspers, L. B. Mokkink, I. M. van der Sluis, C. van den Bos, A. Hartman, F. C. H. Abbink, M. H. van den Berg

**Affiliations:** 1 Emma Children’s Hospital, Amsterdam UMC, Vrije Universiteit Amsterdam, Pediatric Oncology Amsterdam, Amsterdam, The Netherlands; 2 Princess Máxima Center for Pediatric Oncology, Utrecht, The Netherlands; 3 Department of Epidemiology and Biostatistics and Amsterdam Public Health Research Institute, VU University Medical Center, Amsterdam, The Netherlands; 4 Emma Children’s Hospital, Amsterdam UMC, Amsterdam Medical Center, Pediatric Oncology Amsterdam, Amsterdam, The Netherlands; 5 Department of Pediatric Physiotherapy, Erasmus Medical Center Rotterdam/Sophia Children’s Hospital, Rotterdam, The Netherlands

**Keywords:** Neurotoxicity, Diagnostics, Pediatrics, Cancer, Vincristine, Toxicity

## Abstract

**Purpose:**

The aims were to evaluate the construct validity and reliability of the Dutch version of the pediatric-modified Total Neuropathy Score (ped-mTNS) for assessing vincristine-induced peripheral neuropathy (VIPN) in Dutch pediatric oncology patients aged 5–18 years.

**Methods:**

Construct validity (primary aim) of the ped-mTNS was determined by testing hypotheses about expected correlation between scores of the ped-mTNS (range: 0–32) and the Common Terminology Criteria for Adverse Events (CTCAE) (range: 0–18) for patients and healthy controls and by comparing patients and controls regarding their total ped-mTNS scores and the proportion of children identified with VIPN. Inter-rater and intra-rater reliability and measurement error (secondary aims) were assessed in a subgroup of study participants.

**Results:**

Among the 112 children (56 patients and 56 age- and gender-matched healthy controls) evaluated, correlation between CTCAE and ped-mTNS scores was as expected (moderate (*r* = 0.60)). Moreover, as expected, patients had significantly higher ped-mTNS scores and more frequent symptoms of VIPN compared with controls (both *p* < .001). Reliability as measured within the intra-rater group (*n* = 10) (intra-class correlation coefficient (ICC_agreement_) = 0.64, standard error of measurement (SEM_agreement_) = 2.92, and smallest detectable change (SDC_agreement_) = 8.1) and within the inter-rater subgroup (*n* = 10) (ICC_agreement_ = 0.63, SEM_agreement_ = 3.7, and SDC_agreement_ = 10.26) indicates insufficient reliability.

**Conclusion:**

The Dutch version of the ped-mTNS appears to have good construct validity for assessing VIPN in a Dutch pediatric oncology population, whereas reliability appears to be insufficient and measurement error high. To improve standardization of VIPN assessment in children, future research aimed at evaluating and further optimizing the psychometric characteristics of the ped-mTNS is needed.

**Electronic supplementary material:**

The online version of this article (10.1007/s00520-019-05106-3) contains supplementary material, which is available to authorized users.

## Introduction

Vincristine (VCR) is a chemotherapeutic agent which is often used in pediatric oncology for the treatment of various hematological and solid cancers [1]. A frequently occurring side-effect of VCR is peripheral neuropathy [2]. VCR-induced peripheral neuropathy (VIPN) is a mixed sensory, motor and autonomous neuropathy mainly affecting the longer peripheral nerves. Symptoms of VIPN usually start in the distal part of the limbs and may progress proximally [3]. Symptoms include paresthesia, numbness, tingling, loss of proprioception and pain. Rarely, VIPN may lead to profound muscle weakness with symptoms as foot drop and walking difficulties. Autonomous symptoms of VIPN include constipation and dizziness [4]. VIPN showed to adversely affect quality of life in oncology patients [5].

VIPN is a multifactorial toxicity, which is influenced by several determinants. Older children seem to be more affected than younger children, whereas the influence of gender on VIPN is unclear [6]. Moreover, there is a racial difference: Caucasian children tend to have more VIPN than non-Caucasian children [7]. Furthermore, although there is a dose dependent relation between VCR and [8], studies assessing associations between pharmacokinetic parameters of VCR and VIPN report conflicting results [9, 10]. Finally, genetic factors are known to influence susceptibility to VIPN, with several pathways involved such as CEP72 and CYP3A4/5 [8, 11].

Treatment of VIPN is mainly symptomatic using analgesics such as gabapentin, or laxatives in case of constipation. Another treatment option is dose reduction of VCR, although this may lead to suboptimal treatment of the underlying malignancy [12].

In clinical practice, several tools are used to measure VIPN in pediatric oncology patients. The Common Terminology Criteria for Adverse Events (CTCAE) is a tool that assesses the severity of several types of adverse events in oncology patients [13], including those regarding peripheral neuropathy, constipation and neuralgia. Furthermore, Lavoie-Smith et al. [14, 15] developed the Total Neuropathy Score-Pediatric Vincristine (TNS-PV) specifically for the assessment of VIPN in pediatric oncology patients. The TNS-PV consists of a seven-item interview-based questionnaire and a standardized physical examination (testing of vibration and temperature sense, muscle strength and deep tendon reflexes (DTR)) [6]. Besides questionnaires and/or physical examinations, nerve conduction studies in which the conduction velocity of different nerves is measured using somatosensory evoked potentials [16, 17] can also be used to assess VIPN.

However, all of the above-mentioned methods to assess VIPN have some limitations. Although frequently used, the CTCAE shows insufficient sensitivity in detecting motor and sensory neuropathy [2]. Moreover, the TNS-PV can only be used in children aged 6 years or older [14]. Furthermore, nerve conduction studies are invasive, painful, and expensive [14]. Finally, physical examination can only validly be performed and interpreted by specifically trained physicians such as pediatric neurologists [18, 19].

Recently, the pediatric-modified Total Neuropathy Score (ped-mTNS) was developed [20]. This instrument, which consists of an interview-based questionnaire and physical examination, showed to have superior psychometric characteristics compared with other tools for the assessment of peripheral neuropathy in children [2]. Moreover, it is a quick, inexpensive, and non-invasive tool that can be employed by different health care professionals, such as physicians, physical therapists, and nurses [4]. However, the psychometric characteristics of the ped-mTNS have solely been evaluated in North American children with cancer aged 5–18 years.

The primary aim of the current study was to evaluate the construct validity of the Dutch version of the ped-mTNS for the assessment of VIPN in Dutch pediatric oncology patients aged 5–18 years. In addition as secondary aim, reliability of this tool was investigated in a subpopulation.

## Methods

### Study population

The study population of this multicenter cross-sectional study consisted of pediatric oncology patients aged 5–18 years with non- non central nervous system (CNS) malignancies and healthy controls.

Patients were eligible for study participation if they were treated with at least four administrations of at least 1.5 mg/m^2^ (maximum 2 mg) VCR within a period of 6 weeks during treatment of their current malignancy. VIPN assessments were performed within the time frame of at least one month after start of VCR therapy and two months after cessation of VCR therapy maximally. This resulted in the inclusion of patients with the following diagnoses and treatment protocols for the current study: acute lymphoblastic leukemia (ALL) (DCOG ALL-11 protocol [21]), Hodgkin lymphoma (EuroNet-PHL-C1 protocol [22] or C2 protocol [23]), nephroblastoma (SIOP Wilms 2001 protocol [24]), and rhabdomyosarcoma (EpSSG RMS 2005 protocol [25]). VCR was administered either by means of a bolus injection (1–5 min) or a 1-h infusion. The healthy control group consisted of siblings of participating patients and children known to hospital co-workers (relatives or friends). Controls were age- (± 1 year) and gender-matched to the patients on a 1:1 basis. Patients and controls were excluded in case of premorbid developmental disorders, neuromuscular disorders, lower extremity amputations, diabetes mellitus, or in case they were not able to speak or understand the Dutch language. Although strictly speaking the included controls could not develop VIPN (as they did not receive any VCR), hypothetically they may have some degree of peripheral neuropathy. The ped-mTNS is a tool which assesses peripheral neuropathy, either chemotherapy-induced or not, in children. For reasons of clarity, we use the term VIPN in the remainder of this paper, thereby referring to either VCR-induced (patients) or non-VCR-induced (controls) peripheral neuropathy.

### ped-mTNS

The original, English version of the ped-mTNS was translated into Dutch by a non-native English speaker, followed by a translation back to English by a bilingual Dutch-English speaker. Subsequently, this back-translated version was sent to the principal investigator of the original ped-mTNS in the USA [4] in order to have it reviewed and checked by its original developer. Appendix A and B as Supplementary Materials contain the original version and the Dutch version of the ped-mTNS as approved by the developer, respectively.

The questionnaire-part of the ped-mTNS contains eight questions about sensory, functional and autonomic symptoms. These questions were read out aloud by the assessor to the participant. In addition, five different aspects of VIPN were assessed by physical examination: light-touch sensation by Semmes-Weinstein monofilaments (Rolyan–Ability One, Germantown, WI, USA) [4, 26], pin sensibility by Medipin™ (Ltd, Hertfordshire, UK) [4], muscle strength by means of manual muscle testing (graded according to the Medical Research Council guidelines) [4, 27], and DTR of the Achilles and patella (graded according to the Mayo Clinic Criteria) [4, 28]. Due to the unavailability of a Biothesiometer™ (Biomedical Instruments, Newbury, OH, USA) in The Netherlands, the assessment of vibration sense was carried out with a Rydel-Seiffer 64-Hz tuning fork (Gebrueder Martin, Tuttlingen, Germany), which showed to be a valid instrument in the assessment of vibration sense in children [4, 29]. For all items in the questionnaire and the physical examination part, the score ranged between 0 (no symptoms) and 4 (severe symptoms). The worst scores within each of the 3 items as assessed by the questionnaire, together with the scores of the 5 items tested in the physical examinations, are used to calculate the total ped-mTNS score, which is the sum of these 8 scores (range 0 to 32). Children with a total ped-mTNS score of 5 or higher were considered to have VIPN [4].

### CTCAE

The CTCAE (version 4.03) [13] consists of over 330 items scoring adverse events due to cancer treatment divided into 26 different categories. Possible grades range from 0 (no symptoms) and 3 (severe symptoms) or, if the adverse event can be deadly, category 4 is life threatening and category 5 is death. The CTCAE items used for the assessment of VIPN are constipation, peripheral sensory neuropathy, peripheral motor neuropathy, and neuralgia. The maximum CTCAE sum score of these 4 items is 18. Participants with a total score of 2 or higher are considered to have VIPN [6]. The assessor asked to which extent the participants experienced problems as described in the relevant CTCAE items. For the item peripheral sensory neuropathy, additionally DTR of the patella and Achilles were assessed.

### Procedures

Patients were included in Emma Children’s Hospital/Amsterdam University Medical Centers and Sophia Children’s Hospital/Erasmus Medical Center Rotterdam. Study measurements were performed between September 2016 and July 2017. All study participants (patients and controls) were tested once using both the ped-mTNS and CTCAE to assess VIPN. These measurements were performed by the same assessor (SS), who was trained extensively by a pediatric neurologist to perform the VIPN assessments. Furthermore, a subset of randomly selected patients and healthy controls were assessed twice in order to assess intra-rater ((5 patients and 5 controls), both measurements performed by the same assessor) and inter-rater reliability ((7 patients and 3 controls) second measurement performed by a different assessor (MvdV), who was also specifically trained for the study measurements). All patients were measured during regular hospital visits. Healthy controls were measured either during family visits in the hospital or at home. For the assessment of intra-rater reliability, an interval of 4–16 days between the two measurements, without VCR administrations in between, was adhered to.

### Statistics

Assessment of construct validity was determined by calculating the correlation between the ped-mTNS and the CTCAE sum scores in patients as well as the actual differences between these scores. We hypothesized total ped-mTNS and total CTCAE scores to be moderately correlated, since some items between the two systems attempt to measure the same items (peripheral motor neuropathy and peripheral sensory neuropathy), whereas some items are only present in one of the instruments (constipation is only an item in the CTCAE and not in the ped-mTNS). Moreover, we expected to assess a higher correlation between the two measurement tools than the correlation measured in the study of Gilchrist et al. (*r* = 0.07) [2], since in that study the CTCAE scores were retrieved from medical records instead of the prospective assessment of CTCAE scores used in our study. Either the Spearman or Pearson correlation coefficients were calculated, depending on normality of data distribution. According to Nunnally and Bernstein [30], an ICC of > 0.70 was considered as good. Furthermore, a correlation of 0.51–0.69 was considered moderate and < 0.5 as low [31]. We hypothesized ped-mTNS scores of patients to be significantly higher compared with healthy controls and the proportion of patients with VIPN to be significantly higher than those of healthy controls. Differences between mean total ped-mTNS score of patients and controls were analyzed using a Mann-Whitney *U* test. Differences between the proportion of patients and controls who were identified as having VIPN by the ped-mTNS were calculated using a Chi-square test. A two-tailed significance level of 0.05 was used.

Assessment of reliability and standard error of measurement of the ped-mTNS (secondary aims) was carried out as follows. Reliability and measurement error were measured within the inter-rater and intra-rater subgroups by means of intra-class correlation coefficient using the two-way random effects model for agreement (ICC_agreement_) according to the equation ICC_agreement_ =$\frac{{\sigma_p}^2}{{\sigma_p}^2+{\sigma_o}^2+{\sigma_{\mathrm{residual}}}^2}$ where *σ*_error_^2^ = *σ*_*o*_^2^ + *σ*_residual_^2^ [32]. Measurement error was assessed by calculating the standard error of measurement from the same model (i.e., the two-way random effects model for agreement (SEM_agreement_)) by the equation ${\mathrm{SEM}}_{\mathrm{agreement}}=\sqrt{\left({\sigma_o}^2+{\sigma_{\mathrm{residual}}}^2\right)\ }$ and by calculating the smallest detectable change (SDC_agreement_) using the equation ${\mathrm{SDC}}_{\mathrm{agreement}}=1.96\times \sqrt{2}\times {\mathrm{SEM}}_{\mathrm{agreement}}$ [33, 34].

Dichotomized total scores of the ped-mTNS (yes/no VIPN; a total ped-mTNS score of 0–4 indicates no VIPN and a score of ≥ 5 indicates VIPN according to published data by Gilchrist et al. [4]) were used to measure agreement in a 2 × 2 table to calculate positive agreement (PA) (PA = 2*a*/(2*a* + *b* + *c*) and negative agreement (NA) (NA = 2*d*/(2*d* + *b* + *c*).

All results on reliability are reported for patients, healthy controls, and total group separately. All statistics were performed using SPSS for Windows version 22.0 (SPSS, Chicago, IL).

## Results

### Study population

Fifty-six patients and 56 healthy controls (median, interquartile range (IQR) age 9.6 (6.6–14.2) years) were included in the study. Characteristics of the participants are presented in Table 1. The majority of the patients were treated for ALL (*n* = 39, 70%) or Hodgkin lymphoma (*n* = 12, 21%). The mean (standard deviation (SD)) cumulative dose of VCR administered to the patients was 19.6 mg/m^2^ (13.9).

**Table 1:** Baseline characteristics of study participants

	Patients (*n* = 56)^1^	Healthy controls (*n* = 56)^1^
Sex		
Male	32 (57.1)	32 (57.1)
Female	24 (42.9)	24 (42.9)
Age in years (median [IQR])	9.7 [6.3–14.1]	9.6 [7.0–14.4]
Ethnicity		
European	40 (71.4)	39 (69.6)
Middle-Eastern	10 (17.9)	6 (10.7)
African	1 (1.8)	4 (7.1)
Hispanic		4 (7.1)
Other	5 (8.9)	3 (5.4)
Diagnosis^2^		
ALL	39 (69.6)	
Hodgkin lymphoma	12 (21.4)	
Nephroblastoma	1 (1.8)	
Rhabdomyosarcoma	4 (7.1)	
Administration method of VCR^2^		
Bolus injection	47 (83.9)	
1-h infusion	9 (16.1)	
Cumulative dose of VCR (mg/m^2^; mean (SD))^2^	19.6 (13.91)	
Time since last VCR do se (days, median [IQR])^2^	9.5 [0.75–21.0]	

^1^Values represent the number (%) of participants, unless indicated otherwise; ^2^This is only applicable to the patient population

*IQR* interquartile range, *ALL* acute lymphoblastic leukemia, *VCR* vincristine, *SD*: standard deviation

### Construct validity of the ped-mTNS

The correlation between total scores of the ped-mTNS and CTCAE was moderate as expected in patients (*r* = 0.60). Patients had a significantly higher score on ped-mTNS than healthy controls (median (IQR): 10.0 (6.25–13.0) and median (IQR): 0.0 (0.0–1.0), respectively; *p* < 0.001) (Table 2). Furthermore, patients were significantly more often identified as having VIPN (≥ 5 on the ped-mTNS) than healthy controls (86% versus 1.8%, respectively; *p* < 0.001).

**Table 2:** Results of peripheral neuropathy measurements in the two study groups

	Patients (*n* = 56)	Healthy controls (*n* = 56)	*p* value
Total ped-mTNS scores			
Median [IQR]	10.0 [6.25–13.0]	0.0 [0.0–1.0]	< 0.001
Score of 0–4 (*n*, (%))	8 (14.3)	55 (98.2)	
Score 5–32 (*n*, (%))	48 (85.7)	1 (1.8)	
Total CTCAE scores			
Median [IQR]	3 [2–4.75]	0 [0–0]	< 0.001
Score of 0–1 (*n*, (%))	9 (16.1)	55 (98.2)	
Score of 2–4 (*n*, (%))	33 (59.0)	1 (1.8)	
Score of 5–7 (*n*, (%))	12 (21.4)	0 (0)	
Score of 8–18 (*n*, (%))	2 (3.6)	0 (0)	

*ped-mTNS* pediatric-modified Total Neuropathy Score, *IQR* interquartile range *CTCAE* Common Terminology Criteria for Adverse Events

### Reliability

All results regarding reliability for patients, healthy controls, and the total group of participants are depicted in Table 3. The different variance components of the intra-rater reliability group were: *σ*_p_^2^ = 15.3, *σ*_o_^2^ = 0, and *σ*_residual_^2^ = 8.6 and those of the inter-rater reliability were: *σ*_p_^2^ = 23.1, *σ*_o_^2^ = 0, and *σ*_residual_^2^ = 13.7. The ICC_agreement_ of the intra-rater group was 0.64, whereas this was 0.63 of the inter-rater group. Positive agreement was 80%, negative agreement was 83%. Positive agreement and negative agreement of inter-rater reliability were both 100%. Finally, the SEM_agreement_ within the intra-rater group was 2.92 and within the inter-rater group 3.7. The SDC_agreement_ was 8.1 within the intra-rater group and 10.26 within the inter-rater group.

**Table 3:** Reliability outcome measures of the pediatric-modified Total Neuropathy Score

	ICC_agreement_ total ped-mTNS score	95% CI ICC total ped-mTNS score	SDC_agreement_	SEM_agreement_	Positive agreement of total ped-mTNS score	Negative agreement of total ped-mTNS score
Inter-rater reliability patients (*n* = 7)	0.45	− 0.43 to 0.88	12.25	4.42	100%	100%
Inter-rater reliability healthy controls (*n* = 3)	0	-	5.73	2.07	-	100%
Intra-rater reliability patients (*n* = 5)	0	− 1.3 to 0.20	8.96	3.23	80%	0%
Intra-rater reliability healthy controls (*n* = 5)	0	-	0	0	-	100%
Inter-rater reliability total (*n* = 10)	0.63	0.04 to 0.89	10.26	3.7	100%	100%
Intra-rater reliability total (*n* = 10)	0.64	0.06 to 0.90	8.1	2.92	80%	83%

*ICC* intra-class correlation coefficient, *ped-mTNS* pediatric-modified Total Neuropathy Score, *CI* confidence interval, *SDC* smallest detectable change, *SEM* standard error of measurement

## Discussion

In this study, the Dutch version of the ped-mTNS was validated by assessing VIPN in a cohort of Dutch pediatric oncology patients aged 5–18 and age- and gender-matched healthy controls. The correlation between ped-mTNS and CTCAE was found to be as expected (i.e., moderate). Furthermore, patients had significantly higher ped-mTNS scores than controls and were significantly more often identified as having VIPN. These results indicate that this translated version of the ped-mTNS has good construct validity regarding the assessment of VIPN in Dutch pediatric oncology patients. However, reliability of this instrument was insufficient (ICC_agreement_ < 0.7). The outcomes of measurement error showed a SEM_agreement_ of 2.92 and 3.7 and a SDC_agreement_ of 8.1 and 10.26 for the intra-rater and inter-rater subgroups, respectively. Although the minimal important change (MIC) of this instrument is unknown, these SEM_agreement_ and SDC_agreement_ scores appear to be rather high, given the fact that scores can range from 0 to 32 and we used a cutoff value of ≥ 5 to discriminate children with and without VIPN. However, positive agreement and negative agreement were good, with scores between 80 and 100% for intra-rater and inter-rater reliability, respectively.

The results of the current study are comparable to a previous study. Gilchrist et al. [4] recently showed that patients had a significantly higher score on ped-mTNS than healthy controls (mean (SD): 8.7 (4.2); and 1.4 (0.9), respectively). However, in that study, only 9.8% of the controls had a score of 0 on the ped-mTNS, while this percentage was 68% in the current study. This discrepancy can most likely be attributed to differences in carrying out the assessments with the ped-mTNS, such as the level of training of the assessors, and not to a difference in population. The results of Gilchrist et al. showed that healthy controls frequently experienced some disorders of motor function, autonomic symptoms, pin sensation, and distal strength, although in both studies scores of healthy individuals were not high enough to indicate VIPN (ped-mTNS score was < 5) and therefore not of great clinical relevance. In the current study, the healthy control group had a mean (SD) ped-mTNS score of 0.9 (1.5). In the study of Gilchrist et al., similar scores were reported (mean (SD): 1.4 (0.9)). Furthermore, the current study showed an ICC_agreement_ score of the ped-mTNS for intra- and inter-rater reliability of 0.64 and 0.63, respectively. These scores are lower than the ICC sores for intra-rater and inter-rater reliability as reported by Gilchrist et al. (0.99 and 0.98, respectively) [4]. This may be due to the fact that within the study of Gilchrist et al., an interval of only 1 h between two measurements was applied when assessing inter- and intra-rater reliability, whereas in our study, this interval was 4 days minimally. An interval of 1 h may have led to recall bias of the patient for the interviewed questions and of the assessor for the physical examination part, since there is a high chance that the results of the previous measurement are memorized. Furthermore, Gilchrist et al. have used a two-way mixed effect model for consistency for their calculation of ICC (i.e. ICC_consistency_); therefore, the variance due to systematic differences between observers was ignored [32, 33]. By using ICC_agreement_, which we did in our study, this variance of observers was taken into account. However, using ICC_consistency_ instead of ICC_agreement_ leads to higher ICC values, which could be an explanation for the difference in results between these studies [35].

Our study was the first to evaluate psychometric characteristics of a translated version of the ped-mTNS. Our results are important both for clinical purposes and research practice as they contribute to the urgently needed standardization of measuring peripheral neuropathy in children with cancer using high-quality outcome measurement instruments.

This study has some limitations. As previously mentioned, the Rydel-Seiffer 64-Hz tuning fork was used for the assessment of vibration sense instead of the Biothesiometer™, due to unavailability of this device in The Netherlands. However, according to Hilz et al. [29], the Rydel-Seiffer 64-Hz tuning fork is a valid instrument to examine vibration sense and Gilchrist et al. [20] showed a moderate to good correlation (i.e., *r* = − 0.62 to − 0.73, depending on test site) between the Biothesiometer™ and the Rydel-Seiffer 64-Hz tuning fork.

The assessment of reliability and measurement error were only evaluated within a subgroup of 20 participants. Although there is no formal consensus about the minimal number of participants needed to properly asses the inter- and intra-rater reliability [36], the number of participants included for these assessments is probably rather low as can be seen in the 95% confidence intervals of the reliability estimations, due to logistical limitations. Therefore, these results should be interpreted with caution. Future studies assessing the reliability of the ped-mTNS in a larger group of patient are advocated. These studies can be performed within a patient population, since results regarding reliability of healthy controls will probably be difficult to interpret due to floor effects.

Despite certain psychometric limitations, the ped-mTNS is currently considered to be the most optimal instrument for assessing VIPN in children compared with other instruments [37]. Continuous efforts should be made to further improve this instrument, by studying and advancing its reliability and by additionally assessing its validity. Specifically, more research should be undertaken to investigate the content validity of this instrument, by assessing if the ped-mTNS is complete in measuring all the aspects of VIPN in terms of relevance and comprehensibility of this instrument, both for patients and assessor. Finally, it should be investigated to which extent an adapted version of the ped-mTNS could be developed that is suitable for assessing VIPN in children under the age of 5. All above-mentioned efforts may result in a more valid and reliable instrument for the assessment of VIPN in children. Meanwhile, using the current version of the ped-mTNS is advocated since it will lead to more uniformity in assessing VIPN in children with cancer, thereby enabling the comparison of study results for this group of patients [37].

In conclusion, the current study showed that the Dutch translated version of the ped-mTNS has good construct validity, whereas reliability appeared insufficient, although patient numbers for reliability testing were low. In order to improve the comparability of results across different studies investigating VIPN in children, further standardization of VIPN assessment is needed. More research aimed at investigating and improving the quality of the ped-mTNS, or in the development of another instrument to assess VIPN, is needed. This will ultimately lead to a robust instrument and more uniformity in evaluating chemotherapy-induced peripheral neuropathy in children with cancer.

## Electronic supplementary material

ESM 1 (DOCX 14 kb)

ESM 2 (DOCX 14 kb)

## Data Availability

The authors had full control of all primary data and the data that support the findings of this study are available from the corresponding author upon reasonable request.
