# Supplementary material for: Measuring vincristine-induced peripheral neuropathy in children with cancer: validation of the Dutch pediatric–modified Total Neuropathy Score
Source: Support Care Cancer. 2019 Nov 16;28(6):2867–73. doi: 10.1007/s00520-019-05106-3 (PMC7181423; doi:10.1007/s00520-019-05106-3)
Supplement: Supplementary file 1 — (DOCX 14 kb) [file 520_2019_5106_MOESM1_ESM.docx]

**Appendix A. Original version of the Pediatric-modified Total Neuropathy Score**

Sensory Symptoms:____ (record worst score for the three sensations)

“Do you have any parts of your body that are tingly, numb (can hardly feel), or hurt?”

____ Tingly ____ Numb ____ Hurt (record number for each)

If yes, “Where you have those feelings?”

0 None

1 Symptoms limited to fingers or toes

2 Symptoms extend to ankles or wrists

3 Symptoms extend to knee or elbow

4 Symptoms above knee or elbow

Functional Symptoms:____ (record worst score of the three questions)

“Do you have trouble buttoning shirts or zipping zippers?” ____

“Do you have trouble walking such as tripping frequently?” ____

“Do you have trouble going up or down stairs?” ____

If yes to any, “Is it….(read choices)” and record after each question

0 Not Difficult

1 A little difficult

2 Somewhat difficulty

3 I need help

4 I can’t do that at all

Autonomic Symptoms:____ (record worst score of the three questions)

“Do you feel dizzy or light-headed when you get up out of bed?” ____

“Do your hands or feet feel hotter or colder than normal?”

0 Never

1 A little bit

2 Sometimes

3 Very much

4 Almost always

|  | Semmes |  | Semmes |
| --- | --- | --- | --- |
| Toes R |  | Finger R |  |
| L |  | L |  |
| Med Mal R |  | Wrist R |  |
| L |  | L |  |
| Knee R |  | Elb R |  |
| L |  | L |  |

**Clinical Testing** :

Light Touch Sensation: ____

0 Normal

1 Reduced in fingers/toes

2 Reduced up to wrist/ankle

3 Reduced up to elbow/knee

4 Reduce to above elbow/knee

Pin Sensibility: ____

0 Normal

1 Reduced in fingers/toes

2 Reduced up to wrist/ankle

3 Reduced up to elbow/knee

4 Reduce to above elbow/knee

|  | Bioesth |  | Bioesth |
| --- | --- | --- | --- |
| Toes R |  | Finger R |  |
| L |  | L |  |
| Med Mal R |  | Wrist R |  |
| L |  | L |  |
| Knee R |  | Elb R |  |
| L |  | L |  |

Vibration Sensibility:____ (worst score)

0 Normal

1 Reduced in fingers/toes

2 Reduced up to wrist/ankle

3 Reduced up to elbow/knee

4 Reduced to above elbow/knee

Strength: ____ Worst Score (MRC Score R / L)

MRC level: Great Toe ____/____Ankle DF____/____Finger abd____/____ Wrist ext____/____

0 Normal

1 Mild weakness (MRC 4)

2 Moderate weakness (MRC 3)

3 Severe weakness (MRC 2)

4 Paralysis (MRC 1-0)

DTR: ____ (Achilles, Patellar)

0 Normal

1 Ankle reflex reduced (Achilles +1)

2 Ankle reflex absent (Achilles 0, Patellar +2)

3 Ankle reflex absent, others reduced (Achilles 0, Patellar +1)

4 All reflexes absent (all 0)

Total Score: ____/ 32
