# Supplementary material for: Measuring vincristine-induced peripheral neuropathy in children with cancer: validation of the Dutch pediatric–modified Total Neuropathy Score
Source: Support Care Cancer. 2019 Nov 16;28(6):2867–73. doi: 10.1007/s00520-019-05106-3 (PMC7181423; doi:10.1007/s00520-019-05106-3)
Supplement: Supplementary file 2 — (DOCX 14 kb) [file 520_2019_5106_MOESM2_ESM.docx]

**Appendix B. Dutch version of the Pediatric-modified Total Neuropathy Score**

**Interview vragen**

**Vraag sensorische symptomen**

*Zijn er bepaalde lichaamsdelen die tintelen, verdoofd voelen (die je bijna niet kan voelen), of pijn doen?*

____Tintelingen ____Verdoofd ____Pijn (noteer aantal voor elke sensatie)

Zo ja, *Waar heb je dat gevoel?*

0 Nergens

1 Alleen symptomen in vingers of tenen

2 Symptomen uitgebreid tot enkels of polsen

3 Symptomen uitgebreid tot knie of elleboog

4 Symptomen tot boven de knie of elleboog

Score sensorische symptomen:____ (noteer de slechtste score van de drie sensaties)

**Vragen functionele symptomen**

*Heb je problemen met het dichtknopen van een blouse of dichtritsen van je rits? Is dit ………* (lees keuzemogelijkheden voor):

0 Niet moeilijk

1 Een beetje moeilijk

2 Enigszins moeilijk

3 Ik heb hier hulp bij nodig

4 Ik kan dat helemaal niet

*Heb je problemen met lopen? (struikel je bijvoorbeeld vaak?) Is dit ………* (lees keuzemogelijkheden voor):

0 Niet moeilijk

1 Een beetje moeilijk

2 Enigszins moeilijk

3 Ik heb hier hulp bij nodig

4 Ik kan dat helemaal niet

*Heb je problemen met de trap op of af lopen? Is dit ………* (lees keuzemogelijkheden voor):

0 Niet moeilijk

1 Een beetje moeilijk

2 Enigszins moeilijk

3 Ik heb hier hulp bij nodig

4 Ik kan dat helemaal niet

Functionele symptomen:____ (noteer de slechtste score van de drie vragen)

**Vragen autonome symptomen**

*Voel je je duizelig of licht in je hoofd wanneer je opstaat uit bed?*

0 Nooit

1 Een beetje

2 Soms

3 Heel erg

4 Bijna altijd

*Voelen je handen of voeten warmer of kouder aan dan normaal?*

0 Nooit

1 Een beetje

2 Soms

3 Heel erg

4 Bijna altijd

Autonome symptomen:____ (noteer de slechtste score van de twee vragen)

**Neurologisch onderzoek**

|  | Semmes |  | Semmes |
| --- | --- | --- | --- |
| Tenen R |  | Vinger R |  |
| L |  | L |  |
| Med Mal R |  | Pols R |  |
| L |  | L |  |
| Knie R |  | Elleboog R |  |
| L |  | L |  |

Lichte Tast sensatie:____

0 Normaal

1 Verminderd in vingers/tenen

2 Verminderd tot aan de pols/enkel

3 Verminderd tot aan de elleboog/knie

4 Verminderd tot boven de elleboog/knie

Pijnzin:____

0 Normaal

1 Verminderd in vingers/tenen

2 Verminderd tot aan de pols/enkel

3 Verminderd tot aan de elleboog/knie

4 Verminderd tot boven de elleboog/knie

|  | Rydel |  | Rydel |
| --- | --- | --- | --- |
| Tenen R |  | Vinger R |  |
| L |  | L |  |
| Med Mal R |  | Pols R |  |
| L |  | L |  |
| Knie R |  | Elleboog R |  |
| L |  | L |  |

Vibratiezin:____

0 Normaal

1 Verminderd in vingers/tenen

2 Verminderd tot aan de pols/enkel

3 Verminderd tot aan de elleboog/knie

4 Verminderd tot boven de elleboog/knie

Kracht:____ Slechtste Score (Medical Research Council (MRC) Score R / L)

MRC level: Grote teen___/___Enkel dorsoflexie___/___Vinger abductie___/___Pols extensie___/___

0 Normaal

1 Milde zwakte (MRC 4)

2 Matige zwakte (MRC 3)

3 Ernstige zwakte (MRC 2)

4 Paralyse (MRC 1-0)

Reflexen:____ (Achillespees, kniepees)

0 Normaal

1 Verminderde achillespeesreflex (Achilles -1 t/m-3 OF +1)*

2 Afwezige achillespeesreflex (Achilles -4, kniepeesreflex 0 OF Achilles 0, kniepeesreflex +2)*

3 Afwezige achillespeesreflex, verminderde kniepeesreflex (Achilles -4, kniepeesreflex -1 t/m -3 OF Achilles 0 kniepeesreflex +1)*

4 Alle reflexen afwezig (allemaal-4 OF allemaal 0) *

* *Afhankelijk van gebruikte scoringsmethode*
